# Osteoblast and Osteoclast Activity Affect Bone Remodeling Upon Regulation by Mechanical Loading-Induced Leukemia Inhibitory Factor Expression in Osteocytes

**DOI:** 10.3389/fmolb.2020.585056

**Published:** 2020-11-26

**Authors:** Jingke Du, Jiancheng Yang, Zihao He, Junqi Cui, Yiqi Yang, Mingming Xu, Xinhua Qu, Ning Zhao, Mengning Yan, Hanjun Li, Zhifeng Yu

**Affiliations:** ^1^Shanghai Key Laboratory of Orthopedic Implants, Department of Orthopedic Surgery, Shanghai Ninth People’s Hospital, Shanghai Jiao Tong University School of Medicine, Shanghai, China; ^2^Department of Spinal Surgery, People’s Hospital of Longhua Shenzhen, Shenzhen, China; School of Life Sciences, Northwestern Polytechnical University, Xi’an, China; Key Laboratory for Space Bioscience and Biotechnology, Northwestern Polytechnical University, Xi’an, China; ^3^Arthritis Clinic and Research Center, Peking University People’s Hospital, Peking University, Beijing, China; ^4^Department of Pathology, Shanghai Ninth People’s Hospital Affiliated to Shanghai Jiao Tong University School of Medicine, Shanghai, China; ^5^Department of Bone and Joint Surgery, Renji Hospital, School of Medicine, Shanghai Jiao Tong University, Shanghai, China; ^6^Department of Orthodontics, Shanghai Ninth People’s Hospital, Shanghai Jiao Tong University School of Medicine, Shanghai, China

**Keywords:** bone remodeling, osteocytes, leukemia inhibitory factor, osteoblasts, osteoclasts

## Abstract

**Purpose:**

Bone remodeling is affected by mechanical stimulation. Osteocytes are the primary mechanical load-sensing cells in the bone, and can regulate osteoblast and osteoclast activity, thus playing a key role in bone remodeling. Further, bone mass during exercise is also regulated by Leukemia inhibitory factor (LIF). This study aimed to investigate the role of LIF in the mechanical response of the bone, *in vivo* and *in vitro*, and to elucidate the mechanism by which osteocytes secrete LIF to regulate osteoblasts and osteoclasts.

**Methods:**

A tail-suspension (TS) mouse model was used in this study to mimic muscular disuse. ELISA and immunohistochemistry were performed to detect bone and serum LIF levels. Micro-computed tomography (CT) of the mouse femurs was performed to measure three-dimensional bone structure parameters. Fluid shear stress (FSS) and microgravity simulation experiments were performed to study mechanical stress-induced LIF secretion and its resultant effects. Bone marrow macrophages (BMMs) and bone mesenchymal stem cells (BMSCs) were cultured to induce *in vitro* osteoclastogenesis and osteogenesis, respectively.

**Results:**

Micro-CT results showed that TS mice exhibited deteriorated bone microstructure and lower serum LIF expression. LIF secretion by osteocytes was promoted by FSS and was repressed in a microgravity environment. Further experiments showed that LIF could elevate the tartrate-resistant acid phosphatase activity in BMM-derived osteoclasts through the STAT3 signaling pathway. LIF also enhanced alkaline phosphatase staining and osteogenesis-related gene expression during the osteogenic differentiation of BMSCs.

**Conclusion:**

Mechanical loading affected LIF expression levels in osteocytes, thereby altering the balance between osteoclastogenesis and osteogenesis.

## Introduction

Mechanical loading plays an essential role in the maintenance of bone quality and quantity, and osteocytes are critical for sensing mechanical stimulation (Dallas et al., 2013; Bellido, 2014; Choy et al., 2020). *In vivo* studies have shown that osteocytes always exist in a complex mechanical environment, which includes shear stress, tensile strain, and pressure (Iolascon et al., 2013). When stimulated by mechanical strain (Wang et al., 2019), osteocytes can regulate osteoblast proliferation and differentiation by releasing signaling molecules, such as nitric oxide, prostaglandin E2, and adenosine triphosphate (Bakker et al., 2001; Genetos et al., 2005). Studies have shown that bed-ridden patients and astronauts develop osteoporosis due to reduced mechanical bone stimulation, but the mechanism of this phenomenon remains unclear (Norvell et al., 2004; Sibonga, 2013; Yang et al., 2018).

Leukemia inhibitory factor (LIF) belongs to the interleukin (IL)-6 family of cytokines; it has been detected in hypertrophic chondrocytes and vascular sprouts (Grimaud et al., 2002), hematopoietic and (Hilton et al., 1991), osteoblasts (Allan et al., 1990), skeletal (Broholm et al., 2011), and osteocytes (Marusić et al., 1993). Previous studies have shown that muscle loading impact bone development, and LIF influences this process (Broholm et al., 2008). Furthermore, IL-6 expression in osteocytes dramatically increases rats are subjected to mechanical loading-induced stress fractures (Wu et al., 2014), and IL-6 messenger RNA (mRNA) levels are elevated under fluid shear stress (FSS) (Bakker et al., 2014). Hence, we hypothesized that mechanical loading-induced LIF in osteocytes might regulate bone remodeling.

Leukemia inhibitory factor has diverse biological functions; for instance, it can promote proliferation of multiple types of hematopoietic cells, trigger platelet formation, facilitate neuronal survival and formation, and accelerate lipid transport in adipocytes (Metcalf, 2003; Brandt et al., 2015). However, LIF may exhibit contradictory effects in some cells (Nicola and Babon, 2015). Previous reports have shown that LIF has controversial effects on bone mass; some reports suggested that LIF causes a decrease in bone mass, whereas some claimed that LIF causes an increase in bone production (Cornish et al., 1993; Falconi and Aubin, 2007; Nicolaidou et al., 2012; Matsushita et al., 2014). Further, it was also reported that LIF promotes osteoclast differentiation (Richards et al., 2000); however, some studies demonstrated that Fra-2 regulates osteoclast size through LIF/LIF-receptor (LIFR) signaling and hypoxia. In addition, LIF- (Bozec et al., 2008) or LIFR- (Ware et al., 1995) knockout mice exhibit increase in osteoclast numbers and size. In the tail-suspension (TS) model, the hind limbs are free from the weight-bearing load, thus mimicking muscular disuse osteoporosis *in vivo*; thereby providing a suitable approach for elucidating the relationship between mechanical loading and bone metabolism (Morey-Holton and Globus, 1998). Hence, in this study, we aimed to investigate whether osteocytes can regulate bone remodeling by secreting LIF under mechanical stress.

## Materials and Methods

### TS Mouse Model

Two-month-old male C57BL/6J mice were purchased from Shanghai SLAC laboratory animal company (Shanghai, China), and the study was approved by the animal ethics committee of Shanghai ninth people’s hospital, China. All applicable institutional and national guidelines for the care and use of animals were followed. Mice were provided with commercial food and water under specific aseptic (specific pathogen free [SPF]) conditions. Twenty mice were randomly divided into two groups – the TS and control (Ctrl) groups, with ten mice per group. Both groups were maintained in the same environmental conditions. For this study, we applied the TS protocol described by Morey-Holton, with modifications (Morey-Holton and Globus, 1998). Briefly, the tail of each mouse was suspended by applying adhesive tape on its lateral surfaces. Three surgical tapes were then applied circularly at the base, middle, and end of the region to secure the adhesion between the tapes and the tail. The adhesive tape loop at the tip of the tail was passed through a metallic hollow column, which was then connected to a 360° free rotating hook via a metallic wire. The hook was placed on an overhead bar in the middle part of the top of the cage. Thus, these mice were maintained at an approximately 35° head-down tilt, with their hind limbs unloaded. The forelimbs could be used for locomotion. The overall suspension period was 28 days.

### Immunohistochemical

Samples were decalcified in 10% ethylenediaminetetraacetic acid (EDTA) for 3 weeks and embedded in paraffin. For microstructure observation, 4-μm-thick sagittal sections of the medial compartment of the knee joint were cut, and immunohistochemical staining with anti-tartrate-resistant acid phosphatase (TRAP) antibody (ab185716, Abcam, United Kingdom) and anti-LIF antibody (AB-449-NA, R&D Systems, United States) was performed.

### Micro-Computed Tomography (CT) Scanning

At the end of the suspension period, the femurs of the mice were fixed with 4% paraformaldehyde and then maintained in 75% ethanol. Specimens were scanned by micro-CT (μCT 80; Scanco Medical, Zurich, Switzerland), as described previously (Zhou et al., 2019). The micro-CT parameters were as follows: voltage, 70 kV; electric current, 114 μA; and resolution, 10 μm per pixel. The three-dimensional structural parameters, including bone volume fraction (BV/TV), trabecular number (Tb.N), trabecular thickness (Tb.Th), trabecular separation (Tb.Sp), and trabecular bone surface (BS/BV), were analyzed in the same region.

### Enzyme-Linked Immunosorbent Assay (ELISA)

Leukemia inhibitory factor levels in the Ctrl and TS groups were detected using the ELISA kit (MLF00, R&D Systems, United States), according to the manufacturer’s instructions. A microplate reader (Bioteck, Arcugnano [Vicenza], Italy) was used to determine the density OD) of each well. We selected 450 and 540 for wavelength correction.

### Osteocyte Culture and Mechanical Loading

To study osteocytes *in vitro*, we used the osteocyte-like MLO-Y4 cells, which were kindly provided by Dr. Lynda Bonewald (University of Missouri-Kansas City, Kansas City, MO, United States). MLO-Y4 cells were cultured on rat tail collagen type I-coated dishes (Solarbio, Beijing, China) with α-minimum essential medium (α-MEM; Gibco, Grand Island, NY, United States), which contained 5% fetal bovine serum (FBS; Gibco), 5% fetal calf serum (FCS, Gibco), and 1% penicillin/streptomycin (Sigma-Aldrich, St. Louis, MO, United States). The osteocytes were then used for investigating FSS and microgravity loading.

MLO-Y4 cells (2 × 10^5^ cells/slide) were seeded on glass slides (75 mm × 38 mm × 1 mm), which were coated with rat tail collagen type 1 (BD Biosciences, Bedford, MA, United States). They were then cultured (37°C, 5% CO_2_) for 48 h to ensure 80–90% confluence at the time of the flow experiment. FSS was generated using a Streamer^®^ Shear Stress Device (flexercell STR-4000, Flexcell, NC, United States). Some MLO-Y4 cells were exposed to FSS (4 dyne/cm^2^, 5 Hz) for 2 h and then harvested immediately. MLO-Y4 cells in the control group were seeded on the same slides, but were not exposed to FSS.

Microgravity simulation experiments were performed with a random positioning machine (RPM) (made by the Center for Space Science and Applied Research, the Chinese Academy of Sciences, Beijing, China). The RPM was placed in an air incubator at 37°C in 5% CO_2_, and rotation mode of the RPM was set to the random mode at a speed range of 0–10 rpm. MLO-Y4 cells (2 × 10^4^ cells/cm^2^) were seeded in a gas-permeable cell culture disk (Opticell, Nunc) and grown in α-MEM, which contained 5% FBS and 5% FCS, at 37°C in humidified air with 5% CO_2_ for 2 day. Cells were then collected and divided into two groups, the control and RPM groups. After fixing the Opticell disks into the inner frame of RPM and ensuring that there were no air bubbles in the medium, the RPM was rotated randomly at a range of 0–10 rpm for 24 h to simulate microgravity. Cells of the control group were cultured under normal conditions (1G).

### *In vitro* Osteoclastogenesis

Bone marrow macrophages (BMMs) were obtained from the long bones of 4-week-old C57BL/6J mice. Mice were sacrificed, and their femurs and tibias were separated under sterile conditions. Bone marrow was flushed from the mouse femurs and tibias, re−suspended in complete α-MEM, which contained macrophage-colony stimulating factor (M-CSF, 30 ng/mL), and cultured in a 10-cm dish at 37°C in 5% CO_2_. The medium was changed every alternate day to remove the non-adherent cells. At 80% confluence, cells were washed thrice with phosphate-buffered saline (PBS), and then BMMs were collected using trypsin for subsequent experiments. Bone mesenchymal stem cells (BMSCs) were obtained in the same way, but were cultured without M-CSF. For BMM differentiation, BMMs (10^5^ cells/well) were seeded in a 24-well plate and then treated with receptor activator of nuclear factor kappa-B ligand (RANKL, 50 ng/mL) and M-CSF (30 ng/mL) after 12 h, as described in a previous study (Song et al., 2019). We then added LIF (10 ng/mL) to some of the wells and changed the media every alternate day for 7 days until osteoclasts were formed. After fixing the cells with 4% paraformaldehyde for 30 min, anti-TRAP antibody immunohistochemical staining was performed to detect TRAP activity. The Image-Pro Plus software (Media Cybernetics Bethesda, MD, United States) was used to calculate the number of TRAP-positive cells in each well.

### *In vitro* Osteogenesis

After reaching 80–90% density in a 24-well plate, BMSCs were cultured with osteogenesis assay kit (MUBMX-90021, Cyagen, CA, United States) at 37°C in humidified air with 5% CO_2_ for 21 days to induce osteoblasts. Alkaline (ALP) or Alizarin red staining was performed after 14 and 21 days of culture. ALP presence in the cell layers was assessed as follows: the cultured cells were washed thrice with PBS, fixed with 4% paraformaldehyde for 10 min at room temperature (25°C), and then stained using the BCIP/NBT Alkaline Phosphatase Color Development Kit (Beyotime Institute of Biotechnology, Shanghai, China), according to the manufacturer’s instructions. For Alizarin red staining, the cultured cells were fixed with 4% paraformaldehyde for 30 min, and then Alizarin red dye (contained in the MUBMX-90021 kit) was added to the 24-well plate for 3–5 min at room temperature (25°C). The plate was then washed five times with PBS. Semi-quantitative analysis was performed by adding 10% cetylpyridinium chloride (500 μL) (H811089, Macklin, CA, United States) to each well, and absorbance of the supernatant at 562 nm was detected after incubation for 30 min at room temperature.

### Quantitative Reverse-Transcription Polymerase Chain Reaction (qRT-PCR)

Total RNA was extracted using TRIzol reagent (Thermo Scientific, United States) and converted to complementary DNA (cDNA) using the Quantscript RT Kit (Promega, Madison, WI, United States). Next, PCR reactions of the cDNA and SYBR Premix Ex Taq Mix (10 μL) (Selleck, TX, United States) were performed. Real-Time PCR System (Light Cycler 2.0; Roche Diagnostics GmbH, Mannheim, Germany) was employed to determine mRNA levels. The data were analyzed by the 2^–ΔΔ*Ct*^ method, where Ct is threshold cycle. The primer sequences are presented in Table 1.

**TABLE 1:** Primer sequences for the quantitative reverse-transcription polymerase chain reaction.

**Target genes**	**Forward (5′-3′)**	**Reverse (5′-3′)**
β-Actin	AGCCATGTACGTAGCCATCC	CTCTCAGCTGTGGTGGTGAA
Gapdh	ACCCAGAAGACTGTGGATGG	CACATTGGGGGTAGGAACAC
Lif	ATTGTGCCCTTACTGCTGCTG	GCCAGTTGATTCTTGATCTGGT
Bad	AAGTCCGATCCCGGAATCC	GCTCACTCGGCTCAAACTCT
Bax	TGAAGACAGGGGCCTTTTTG	AATTCGCCGGAGACACTCG
Ocn	CTGACCTCACAGATCCCAAGC	TGGTCTGATAGCTCGTCACAAG
Alp	GCCTGGATCTCATCAGTATTTGG	GTTCAGTGCGGTTCCAGACAT
Runx2	CCGGGAATGATGAGAACTA	ACCGTCCACTGTCACTTT
Osteorix	CTCTCTGCTTGAGGAAGAAG	GTCCATTGGTGCTTGAGAAG
Opg	ACCCAGAAACTGGTCATCAGC	CTGCAATACACACACTCATCACT
Traf6	AAACCACGAAGAGGTCATGG	GCGGGTAGAGACTTCACAGC
Ctsk	GGACCCATCTCTGTGTCCAT	CCGAGCCAAGAGAGCATATC
Dcst	AAAACCCTTGGGCTGTTCTT	AATCATGGACGACTCCTTGG
c-Fos	CCAGTCAAGAGCATCAGCAA	AAGTAGTGCAGCCCGGAGTA

### Western Blot (WB) Analysis

To separate differently sized proteins in the samples, sodium dodecylsulfate-polyacrylamide gel (10% concentration) electrophoresis was performed. The proteins were then transferred onto polyvinylidene difluoride (PVDF) membranes (Millipore, Billerica, MA, United States). The PVDF membranes were blocked using 5% non-fat milk for 30 min and then incubated with primary antibodies against STAT3 (1:1,000; Cell Signaling Technology, MA, United States), p-STAT3 (1:1,000; Cell Signaling Technology), ERK (1:1,000; Cell Signaling Technology), p-ERK (1:2,000; Cell Signaling Technology), and β-actin (1:1,000; Cell Signaling Technology) at 4°C overnight. The membranes were washed four times with tris-buffered saline with Tween 20, and then incubated with horseradish peroxidase-conjugated secondary antibodies (1:10,000; Laizee Biotech, Shanghai, China) at room temperature for 1 h. Chemiluminescence detection was performed using an enhanced chemiluminescence Kit (Beyotime Institute of Biotechnology) and an Omega Lum^TM^ C Imaging System (Gel Company, San Francisco, CA, United States) (Han et al., 2018). The relative intensity of the bands is quantified by ImageJ.

### Cell Viability Assay

MLO-Y4 cells (10000/cm^2^) were seeded in 96-well plates, and some of them were treated with LIF (10 ng/mL). The cells were then incubated for 24 h. CCK-8 (C6005, NCM Biotech, United States) was added to the wells to obtain a final concentration of 10%, and incubated at 37°C for 2 h. The microplate reader (Bioteck, Arcugnano [Vicenza], Italy) was used to detect absorbance at 450 nm (Liu et al., 2017).

### Microarray Analysis

Three independent biological replicates of the FSS and Ctrl groups were used to perform microarray analysis. Briefly, after FSS treatment, MLO-Y4 cells were dissolved in TRIzol and sent for microarray analysis to Oebiotech (Shanghai, China^1^). Construction of an Illumina library and sequencing on an Illumina His Eq. 2000 platform were conducted by Oebiotech.

### Statistical Analysis

Statistical analyses of data were performed using GraphPad Prism 5.0 (GraphPad Software Inc., CA, United States). Each experiment was repeated at least thrice. All quantitative values were presented as the mean ± standard deviation (SD), and the differences were evaluated by one−way analysis of variance (ANOVA) or Student’s *t*-test, followed by Bonferroni correction for multiple comparisons. *P* < 0.05 was considered statistically significant.

## Results

**FIGURE 1:**
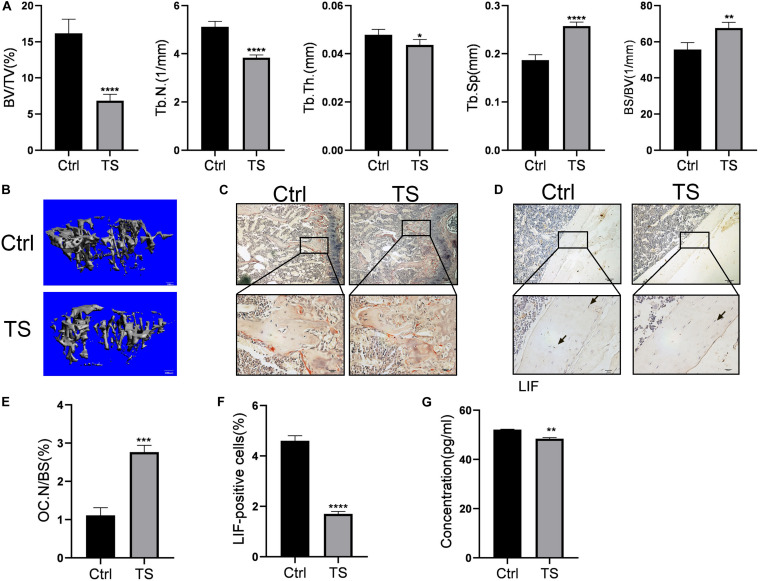
Micro-CT analysis of Tail-suspension mice **(A)** Quantitative analysis of BV/TV, Tb.N, Tb.Th, BS/BV and Tb.Sp. **(B)** Micro-CT 3D reconstruction pictures. **(C)** Osteoclasts in the sub-growth-plate bone of tibia were stained using tartrate-resistant acid phosphatase. **(D)** The expression of LIF in tibias was detected by immunohistochemical staining. **(E)** Osteoclast numbers in Figure 1C were counted. **(F)** LIF positive cells in Figure 1D were counted. **(F)** Expression level of LIF in the serum was detected by ELISA. Black arrow: LIF positive osteocyte. All data are shown as means ± standard deviations (*n* = 10), *p < 0.05, **p < 0.01, ***p < 0.005 and ****p < 0.001.

At the end of the suspension period, femurs of the TS mice showed low bone mass. Bone volume/total volume(BV/TV), trabecular number(Tb.N), trabecular thickness(Tb.Th), trabecular separation(Tb.Sp), and bone surface/bone volume(BS/BV) (Figure 1A) were decreased and osteoclast number(OC.N/BS) (Figures 1C,E) was increased in the TS group, compared to the values in the control group. Micro-CT 3D reconstruction pictures are shown in Figure 1B. Additionally, TS mice showed lower LIF expression levels in both bone tissues and serum, as detected by immunohistochemistry (Figure 1D) and ELISA (Figure 1G), respectively. Quantitative analysis of immunohistochemistry is shown in Figure 1F.

**FIGURE 2:**
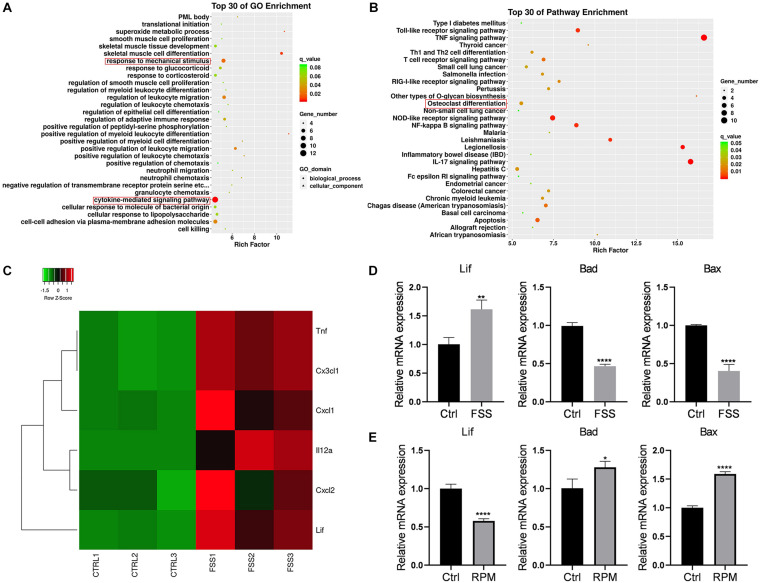
LIF expression in the FSS or microgravity-treated MLO-Y4 cells **(A)** GO and **(B)** KEGG pathway enrichment analyses were performed. **(C)** Heatmap of the expression levels of various cytokines based on the RNA sequencing results is shown. **(D)** LIF expression was increased under FSS, as determined by RT-PCR. **(E)** LIF expression was reduced in the microgravity environment, as determined by RT-PCR. All data are shown as means ± standard deviations (*n* = 3), *p < 0.05, **p < 0.01, ***p < 0.005 and ****p < 0.001.

RNA sequencing was conducted for the FSS-treated and control MLO-Y4 cells. After performing Gene Ontology (GO) (Figure 2A) and Kyoto Encyclopedia of Genes and Genomes (KEGG) pathway (Figure 2B) enrichment analyses, we found that differential genes were enriched in the cytokine-related pathways. According to the RNA sequencing results, the expression levels of various cytokines, including LIF, CXCL1, and CXCL2, were altered (Figure 2C). Thus, we validated a series of cytokine expression levels by qRT-PCR (Figure 2D) and confirmed that LIF expression increased on FSS treatment. This finding was consistent with the RNA sequencing results. Simultaneously, pro-apoptotic genes, such as BAX and BAD, were downregulated after FSS treatment, indicating that FSS exposure possibly prevented MLO-Y4 cell apoptosis. Meanwhile, MLO-Y4 cells were cultivated in a microgravity environment after RPM treatment for 24 h, and the LIF gene expression levels were observed to decrease dramatically with the increase in gene expression levels of apoptosis-related genes (Figure 2E).

**FIGURE 3:**
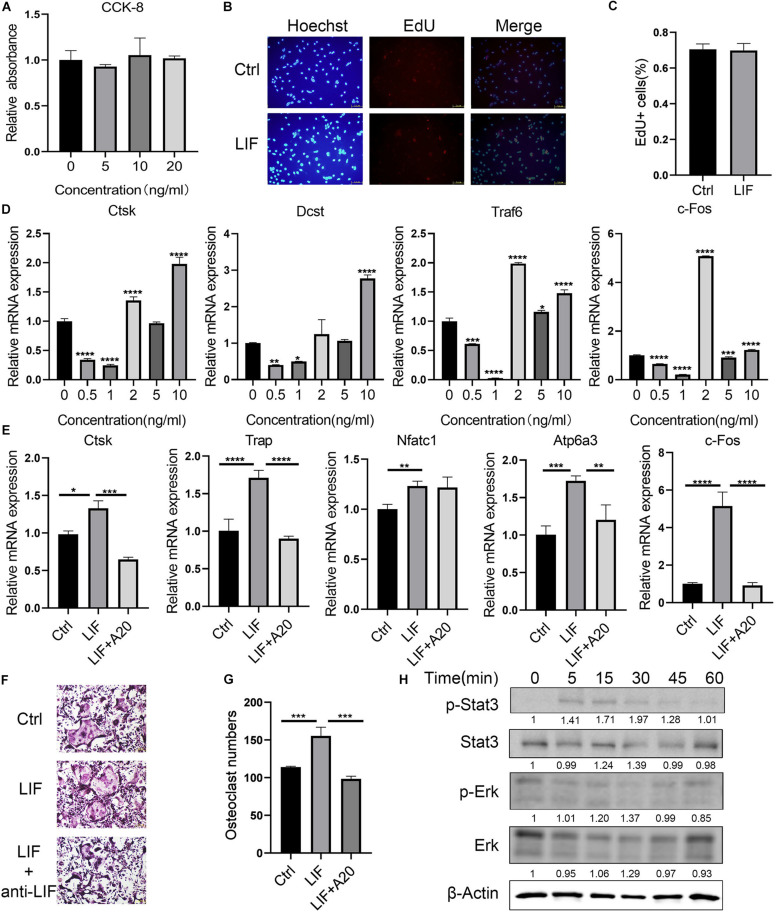
LIF promotes BMM differentiation into osteoclasts **(A)** LIF, at different concentrations, did not affect BMM proliferation, as shown by the OD value calculated during CCK-8 analysis. **(B)** BMM cells were treated with LIF (10 ng/mL) for 24 h, and then EdU assay was performed. **(C)** EdU-positive cells were counted. **(D)** BMM cells were treated with LIF at different concentrations, and then osteoclast-related genes were detected. **(E)** RT-PCR results showed that LIF promoted osteoclast-related gene expression. **(F)** Representative images of TRAP staining of osteoclasts are shown. **(G)** The number of osteoclasts in each well (96-well plate) and all the cells with ≥3 nuclei were counted. **(H)** After treatment with LIF at different times, STAT3 and p-STAT3 protein levels in BMMs were determined by western blot analysis. All data are shown as the mean ± SD (*n* = 3); *p < 0.05, **p < 0.01, ***p < 0.005 and ****p < 0.001.

Bone marrow macrophages were treated with LIF at different concentrations, and then CCK-8 (Figure 3A) and EdU (Figures 3B,C) assays were performed. Results of both assays showed that LIF had no significant influence on BMM cell viability. Verify the effects of LIF on osteoclastogenesis, different concentrations of LIF were added to the BMMs during their differentiation process. As shown in Figure 3D, when LIF concentration was >2 ng/mL, especially at concentration 10 ng/mL, expression of osteoclast-related genes was enhanced. Thus, 10 ng/mL was chosen as the final experimental concentration. Then, BMMs were treated with M−CSF (30 ng/mL), RANKL (50 ng/mL), and LIF (10 ng/mL), with or without LIF-neutralizing (20 ng/mL), until mature osteoclasts were formed. Numerous TRAP-positive multinucleated osteoclasts, formed in the three groups of treated BMMs, are shown in Figure 3F. The results (Figure 3G) showed more osteoclasts in the LIF-treated group than those in the control group, and this could be reversed by using LIF-neutralizing. Osteoclast-related gene expression levels (Figure 3E) also indicated that LIF promoted osteoclast formation, but LIF-neutralizing moderated this facilitation function of LIF. After starvation for 3 h, protein samples of the LIF-treated BMMs were extracted at different times. Figure 3H shows that after treatment for 5 min, LIF promoted STAT3 phosphorylation, but had no effects on ERK. These results showed that LIF promoted BMM differentiation via the STAT3 signaling pathway.

**FIGURE 4:**
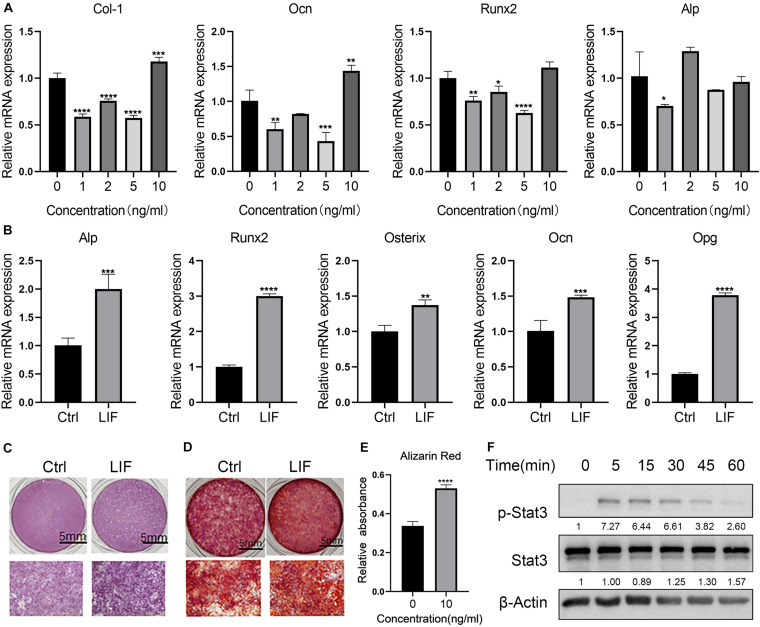
LIF promotes the osteogenesis of BMSC **(A)** after treated with different concentration of LIF, osteogenesis-related genes were detected by RT-PCR. **(B)** After 14 days of culture, osteogenesis-related gene expression levels were tested using RT-PCR. **(C)** Representative ALP staining image of BMSC after being cultured 7 days in the osteogenic medium. **(D)** Alizarin red staining was performed after 21 days. **(E)** Semiquantitative analysis of Alizarin red staining. **(F)** Western blot results of BMSCs after being treated with LIF at different times. All data are shown as means ± standard deviations (*n* = 3), *p < 0.05, **p < 0.01, ***p < 0.005 and ****p < 0.001.

To detect the best concentration of LIF for osteogenesis using the osteogenesis assay kit, LIF was added at different concentrations to the BMSCs. Osteogenesis-related genes were tested on day 14. Consistent with osteoclastogenesis, osteogenesis was greatly promoted by LIF (10 ng/mL) (Figure 4A). As shown in Figure 4B, LIF promoted osteogenesis-related genes, including RUNX2, OCN, and ALP. After 14- and 21-day differentiation culture, BMSCs were stained with ALP (Figure 4C) or Alizarin red (Figure 4D). Images showed that the LIF-treated group presented more ALP- and Alizarin-positive nodules, and this finding was consistent with the results of semi-quantitative analysis of Alizarin red staining (Figure 4E). Western blot analysis showed that LIF activated the STAT3 signaling pathway in the BMSCs (Figure 4F), indicating that LIF exerted influence BMSCs through the STAT3 signaling pathway.

## Discussion

In this study, the TS model was adopted to mimic muscular disuse *in vivo* and investigate the relationship between mechanical stimulation and bone quality. At the end of the tail suspension period, BV/TV, Tb.N, and Tb.Th of the TS mice were decreased significantly. Consistent with our findings, it was reported that hind limb unloading promotes osteocyte apoptosis and decreases bone mass (Swift et al., 2010; Cabahug-Zuckerman et al., 2016).

Both FSS exposure and a microgravity environment can affect LIF expression levels. Previous reports showed the effects of LIF on muscle load and exercise, further influencing bone mineral mass (Rauch and Schoenau, 2001; Brotto and Bonewald, 2015; Jia et al., 2018). Liang et al. (2011) found that continuous orthodontic forces promoted LIF/LIFR expression in the periodontal tissue. These findings showed that LIF plays a role in the mechanical response of the bone. Besides, evidence on the promotion of the anabolic response of bones by low-magnitude mechanical stimulation has also been reported (Ozcivici et al., 2010). Additionally, studies have shown that LIF can promote multinucleated giant cell number and nucleation (Gouin et al., 1999). In our study, LIF (10 ng/mL) was co-cultured with BMMs to investigate whether LIF affected osteoclast formation, indicating greater differentiation ability of LIF. However, when anti-LIF antibody was added to the osteoclastogenesis medium, LIF auxo-action was moderated, demonstrating that LIF could promote BMM differentiation. Several signaling pathways have been shown to be related to osteoclast differentiation and function. Many reports show that STAT3 drives NFATc1 transcription and osteoclast differentiation (Yang et al., 2019), indicating that LIF affects osteoclastogenesis by promoting STAT3 phosphorylation.

Metcalf and Gearing (1989) reported that LIF overexpression *in vivo* increases trabecular bone mass. In our study, during BMSC differentiation, ALP staining and RT-PCR results in the first 14 days demonstrated that LIF promoted osteogenesis, and this finding is consistent with previously reported data (Poulton et al., 2012). STAT3 phosphorylation level in BMSCs was determined after LIF (10 ng/mL) treatment, and it was found that LIF influenced BMSCs via the same signaling pathway (STAT3 signaling pathway).

Nevertheless, there are some limitations of our study. We observed that FSS promoted LIF expression, but the mechanism of this phenomenon remains unknown. In this study, a TS model corresponding to the microgravity experiment *in vitro* is designed, but osteocytes in TS mice are not in a microgravity environment. Besides, we tested LIF function only in BMSCs, whether LIF affected other cell lines, such as 3T3-E1, remains unknown. Last, we tested only the phosphorylation levels of STAT3, and not the downstream pathway, which could partly explain why exercise promoted bone mass increase. The promoting effects of LIF on the two cell lines used in this study showed that it possibly affected bone reconstruction and the decrease in its expression levels resulted in the reduction of bone renewal ability. This, in turn, resulted in poor replacement of the old bone by the new one.

In conclusion, our study demonstrated that mechanical stress could stimulate LIF expression in osteocytes. Besides, LIF simultaneously promoted osteoclastogenesis and osteogenesis. Mechanistically, LIF could increase the phosphorylation levels of STAT3, which is related to osteoclast and osteoblast formation.

## Data Availability Statement

The datasets presented in this study can be found in online repositories. The names of the repository/repositories and accession number(s) can be found in the article/supplementary material.

## Ethics Statement

The animal study was reviewed and approved by Animal Ethics Committee of Shanghai Ninth People’s Hospital, China.

## Author Contributions

All authors listed have made a substantial, direct and intellectual contribution to the work, and approved it for publication.

## Conflict of Interest

The authors declare that the research was conducted in the absence of any commercial or financial relationships that could be construed as a potential conflict of interest.

## Footnotes

1 https://www.oebiotech.com/

## References

[B1] Allan E. H. Hilton J. D. Brown A. M. Evely S. R. Yumita S. Metcalf D. (1990). Osteoblasts display receptors for and responses to leukemia-inhibitory factor. *J. Cell. Physiol.* 145 110–119. 10.1002/jcp.1041450116 2170427

[B2] Bakker A. D. Kulkarni R. N. Klein-Nulend J. Lems W. F. (2014). IL-6 alters osteocyte signaling toward osteoblasts but not osteoclasts. *J. Dent. Res.* 93 394–399. 10.1177/0022034514522485 24492932

[B3] Bakker A. D. Soejima K. Klein-Nulend J. Burger E. H. (2001). The production of nitric oxide and prostaglandin E(2) by primary bone cells is shear stress dependent. *J. Biomech.* 34 671–677. 10.1016/s0021-9290(00)00231-1 11311708

[B4] Bellido T. (2014). Osteocyte-driven bone remodeling. *Calcif. Tissue Int.* 94 25–34. 10.1007/s00223-013-9774-y 24002178 PMC3947228

[B5] Bozec A. Bakiri L. Hoebertz A. Eferl R. Schilling A. F. Komnenovic K. (2008). Osteoclast size is controlled by Fra-2 through LIF/LIF-receptor signalling and hypoxia. *Nature* 454 221–225. 10.1038/nature07019 18548006

[B6] Brandt N. O’Neill H. M. Kleinert M. Schjerling P. Vernet E. Steinberg G. R. (2015). Leukemia inhibitory factor increases glucose uptake in mouse skeletal muscle. *Am. J. Physiol. Endocrinol. Metab.* 309 E142–E153. 25968579 10.1152/ajpendo.00313.2014

[B7] Broholm C. Laye J. M. Brandt C. Vadalasetty R. Pilegaard H. Pedersen K. B. (2011). LIF is a contraction-induced myokine stimulating human myocyte proliferation. *J. Appl. Physiol. (1985)* 111 251–259. 10.1152/japplphysiol.01399.2010 21527666

[B8] Broholm C. Mortensen O. H. Nielsen S. Akerstrom T. Zankari A. Dahl B. (2008). Exercise induces expression of leukaemia inhibitory factor in human skeletal muscle. *J. Physiol.* 586 2195–2201. 10.1113/jphysiol.2007.149781 18292129 PMC2465206

[B9] Brotto M. Bonewald L. (2015). Bone and muscle: Interactions beyond mechanical. *Bone* 80 109–114. 10.1016/j.bone.2015.02.010 26453500 PMC4600532

[B10] Cabahug-Zuckerman P. Frikha-Benayed D. Majeska R. J. Tuthill A. Yakar S. Judex S. (2016). Osteocyte apoptosis caused by hindlimb unloading is required to trigger osteocyte rankl production and subsequent resorption of cortical and trabecular bone in mice femurs. *J. Bone Miner. Res.* 31 1356–1365. 10.1002/jbmr.2807 26852281 PMC5488280

[B11] Choy M. H. V. Wong Y. M. R. Chow H. K. S. Li C. M. Chim N. Y. Li K. T. (2020). How much do we know about the role of osteocytes in different phases of fracture healing? A systematic review. J. Orthop. Translat. 21 111–121. 10.1016/j.jot.2019.07.005 32309136 PMC7152791

[B12] Cornish J. Callon K. King A. Edgar S. Reid I. R. (1993). The effect of leukemia inhibitory factor on bone *in vivo*. *Endocrinology* 132 1359–1366. 10.1210/endo.132.3.8440191 8440191

[B13] Dallas S. L. Prideaux M. Bonewald L. F. (2013). The osteocyte: an endocrine cell and more. *Endocr. Rev.* 34 658–690. 10.1210/er.2012-1026 23612223 PMC3785641

[B14] Falconi D. Aubin J. E. (2007). LIF inhibits osteoblast differentiation at least in part by regulation of HAS2 and its product hyaluronan. *J. Bone Miner. Res.* 22 1289–1300. 10.1359/jbmr.070417 17451373

[B15] Genetos D. C. Geist J. G. Liu D. Donahue J. H. Duncan L. R. (2005). Fluid shear-induced ATP secretion mediates prostaglandin release in MC3T3-E1 osteoblasts. *J. Bone Miner. Res.* 20 41–49. 10.1359/jbmr.041009 15619668 PMC2929123

[B16] Gouin F. Couillaud S. Cottrel M. Godard A. Passuti N. Heymann D. (1999). Presence of leukaemia inhibitory factor (LIF) and LIF-receptor chain (gp190) in osteoclast-like cells cultured from human giant cell tumour of bone. Ultrastructural distribution. Cytokine 11 282–289. 10.1006/cyto.1998.0429 10328867

[B17] Grimaud E. Blanchard F. Charrier C. Gouin F. Redini F. Heymann D. (2002). Leukaemia inhibitory factor (lif) is expressed in hypertrophic chondrocytes and vascular sprouts during osteogenesis. *Cytokine* 20 224–230. 10.1006/cyto.2002.2002 12550107

[B18] Han J. Gao W. Su D. Liu Y. (2018). Gypenoside inhibits RANKL-induced osteoclastogenesis by regulating NF-kappaB, AKT, and MAPK signaling pathways. *J. Cell. Biochem.* 119 7310–7318. 10.1002/jcb.27028 29797602

[B19] Hilton D. J. Nicola N. A. Metcalf D. (1991). Distribution and comparison of receptors for leukemia inhibitory factor on murine hemopoietic and hepatic cells. *J. Cell. Physiol.* 146 207–215. 10.1002/jcp.1041460204 1900303

[B20] Iolascon G. Resmini G. Tarantino U. (2013). Mechanobiology of bone. *Aging Clin. Exp. Res.* 25(Suppl. 1), S3–S7. 24046028 10.1007/s40520-013-0101-2

[B21] Jia D. Cai M. Xi Y. Du S. ZhenjunTian (2018). Interval exercise training increases LIF expression and prevents myocardial infarction-induced skeletal muscle atrophy in rats. *Life Sci.* 193 77–86. 10.1016/j.lfs.2017.12.009 29223542

[B22] Liang Y. Zhou Y. Jiang T. Zhang Z. Wang S. Wang Y. (2011). Expression of LIF and LIFR in periodontal tissue during orthodontic tooth movement. *Angle Orthod.* 81 600–608. 10.2319/102510-622.1 21446866 PMC8919751

[B23] Liu X. Chin J.-F. Qu X. Bi H. Liu Y. Yu Z. (2017). The beneficial effect of praeruptorin C on osteoporotic bone in ovariectomized mice via suppression of osteoclast formation and bone Resorption. *Front. Pharmacol.* 8:627. 10.3389/fphar.2017.00627 28955232 PMC5601062

[B24] Marusić A. Kalinowski F. J. Jastrzebski S. Lorenzo A. J. (1993). Production of leukemia inhibitory factor mRNA and protein by malignant and immortalized bone cells. *J. Bone Miner. Res.* 8 617–624. 10.1002/jbmr.5650080513 8511989

[B25] Matsushita K. Itoh S. Ikeda S. Yamamoto Y. Yamauchi Y. Hayashi M. (2014). LIF/STAT3/SOCS3 signaling pathway in murine bone marrow stromal cells suppresses osteoblast differentiation. *J. Cell. Biochem.* 115 1262–1268. 10.1002/jcb.24777 24464633

[B26] Metcalf D. (2003). The unsolved enigmas of leukemia inhibitory factor. *Stem Cell.* 21 5–14. 10.1634/stemcells.21-1-5 12529546

[B27] Metcalf D. Gearing D. P. (1989). Fatal syndrome in mice engrafted with cells producing high levels of the leukemia inhibitory factor. *Proc. Natl. Acad. Sci. U. S. A.* 86 5948–5952. 10.1073/pnas.86.15.5948 2569739 PMC297748

[B28] Morey-Holton E. R. Globus R. K. (1998). Hindlimb unloading of growing rats: a model for predicting skeletal changes during space flight. *Bone* 22 (5 Suppl.), 83S–88S. 9600759 10.1016/s8756-3282(98)00019-2

[B29] Nicola N. A. Babon J. J. (2015). Leukemia inhibitory factor (LIF). *Cytokine Growth Factor Rev.* 26 533–544. 26187859 10.1016/j.cytogfr.2015.07.001 PMC4581962

[B30] Nicolaidou V. Wong M. M. Redpath A. N. Ersek A. Baban D. F. Williams L. M. (2012). Monocytes induce STAT3 activation in human mesenchymal stem cells to promote osteoblast formation. *PLoS One* 7:e39871. 10.1371/journal.pone.0039871 22802946 PMC3389003

[B31] Norvell S. M. Alvarez M. Bidwell P. J. Pavalko M. F. (2004). Fluid shear stress induces beta-catenin signaling in osteoblasts. *Calcif. Tissue Int.* 75 396–404. 10.1007/s00223-004-0213-y 15592796

[B32] Ozcivici E. Luu Y. K. Adler B. Qin Y.-X. Rubin J. Judex S. (2010). Mechanical signals as anabolic agents in bone. *Nat. Rev. Rheumatol.* 6 50–59. 10.1038/nrrheum.2009.239 20046206 PMC3743048

[B33] Poulton I. J. McGregor N. Pompolo S. Walker E. C. Sims N. A. (2012). Contrasting roles of leukemia inhibitory factor in murine bone development and remodeling involve region-specific changes in vascularization. *J. Bone Miner. Res.* 27 586–595. 10.1002/jbmr.1485 22143976

[B34] Rauch F. Schoenau E. (2001). The developing bone: slave or master of its cells and molecules? *Pediatr. Res.* 50 309–314. 10.1203/00006450-200109000-00003 11518815

[B35] Richards C. D. Langdon C. Deschamps P. Pennica D. Shaughnessy S. G. (2000). Stimulation of osteoclast differentiation in vitro by mouse oncostatin M, leukaemia inhibitory factor, cardiotrophin-1 and interleukin 6: synergy with dexamethasone. *Cytokine* 12 613–621. 10.1006/cyto.1999.0635 10843736

[B36] Sibonga J. D. (2013). Spaceflight-induced bone loss: is there an osteoporosis risk? *Curr. Osteoporos. Rep.* 11 92–98. 10.1007/s11914-013-0136-5 23564190

[B37] Song C. Yang X. Lei Y. Zhang Z. Smith W. Yan J. (2019). Evaluation of efficacy on RANKL induced osteoclast from RAW264.7 cells. *J. Cell. Physiol.* 234 11969–11975. 10.1002/jcp.27852 30515780

[B38] Swift J. M. Nilsson M. I. Hogan H. A. Sumner L. R. Bloomfield S. A. (2010). Simulated resistance training during hindlimb unloading abolishes disuse bone loss and maintains muscle strength. *J. Bone Miner. Res.* 25 564–574. 10.1359/jbmr.090811 19653816

[B39] Wang T. Yang L. Jiang J. Liu Y. Fan Z. Zhong C. (2019). Pulsed electromagnetic fields: promising treatment for osteoporosis. *Osteoporos. Int.* 30 267–276. 10.1007/s00198-018-04822-6 30603841

[B40] Ware C. B. Horowitz M. C. Renshaw B. R. Hunt J. S. Liggitt D. Koblar S. A. (1995). Targeted disruption of the low-affinity leukemia inhibitory factor receptor gene causes placental, skeletal, neural and metabolic defects and results in perinatal death. *Development* 121 1283–1299. 7789261 10.1242/dev.121.5.1283

[B41] Wu A. C. Kidd L. J. Cowling N. R. Kelly W. L. Forwood M. R. (2014). Osteocyte expression of caspase-3, COX-2, IL-6 and sclerostin are spatially and temporally associated following stress fracture initiation. *Bonekey Rep.* 3 571. 10.1038/bonekey.2014.66 PMC4162464 25228984

[B42] Yang J. Zhang G. Dong D. Shang P. (2018). Effects of iron overload and oxidative damage on the musculoskeletal system in the space environment: data from spaceflights and ground-based simulation models. *Int. J. Mol. Sci.* 19:2608. 10.3390/ijms19092608 30177626 PMC6163331

[B43] Yang Y. Chung M. R. Zhou S. Gong X. Xu H. Hong Y. (2019). STAT3 controls osteoclast differentiation and bone homeostasis by regulating NFATc1 transcription. *J. Biol. Chem.* 294 15395–15407. 10.1074/jbc.ra119.010139 31462535 PMC6802509

[B44] Zhou F. Mei J. Yuan K. Han X. Qiao H. Tang T. (2019). Isorhamnetin attenuates osteoarthritis by inhibiting osteoclastogenesis and protecting chondrocytes through modulating reactive oxygen species homeostasis. *J. Cell. Mol. Med.* 23 4395–4407. 10.1111/jcmm.14333 30983153 PMC6533508

